# Retrospective comparison study of warfarinised trauma patients and an age-matched control group of nonwarfarinised patients

**DOI:** 10.1186/cc11041

**Published:** 2012-03-20

**Authors:** M Omar, P Stevens, T Jenkins, K Morris, H Hussain, A Evans

**Affiliations:** 1 Morriston Hospital, Swansea, UK

## Introduction

There are several studies stating the association of mortality between trauma and anticoagulation; however, it is difficult to ascertain a credible conclusion due to the small number of data and inconclusive results. Some studies have showed a significant increased risk of morbidity and mortality. We analysed retrospective data of 45,798 trauma patients, out of which 254 were on warfarin. The incidence of death continues to rise and there are no specific strategies to reduce haemodilution and coagulopathy which maybe the underlying cause of mortality.

## Methods

A retrospective analysis of a national database collected in 2009 and 2010, from the Trauma Audit and Research Network (TARN) UK. The data also contain vital information including age, Glasgow Coma Scale (GSC), Injury Severity Score (ISS), INR, blood products given, number of days in hospital and clinical outcome. We evaluated trauma patients who were on warfarin therapy and compared their clinical outcome and mortality to age-matched patients with similar injuries not on warfarin.

## Results

A total of 45,780 adult patients were analysed. These were subdivided into 32,225 young patients under 65 years with median age 60.5, of which 59 were on warfarin; and 13,555 older patients aged over 65 with median age 80.4, of which 195 were on warfarin. The mortality rate in warfarinised patients was significantly higher than in the nonwarfarinised age-matched group aged <65 (5/59, 8.5% vs. 1,223/32,163, 3.8%; *P *< 0.001; 95% CI). The group age >65 included 13,555, of which 195 were warfarinised (4.7/195, 24.1% vs. 1,501/13,360, 11.3%; *P *< 0.001; 95% CI).

## Conclusion

This data analysis proves that mortality is significantly higher in warfarinised patients compared to the nonwarfarinised age-matched group. Future research needs to focus on both developing a practical procedure reducing risks of morbidity and mortality by exploring coagulopathy and early correction of coagulations. Anticoagulated patients are more likely to receive aggressive i.v. fluid resuscitations as the result of haemorrhage which leads to haemodilution and further exacerbates coagulopathy. This cascade of events is the underlying mechanism causative to mortality.
